# Assessment of Roles for Calreticulin in the Cross-Presentation of Soluble and Bead-Associated Antigens

**DOI:** 10.1371/journal.pone.0041727

**Published:** 2012-07-27

**Authors:** Natasha Del Cid, Lianjun Shen, Janice BelleIsle, Malini Raghavan

**Affiliations:** 1 Graduate Program in Immunology, University of Michigan Medical School, Ann Arbor, Michigan, United States of America; 2 Department of Microbiology and Immunology, University of Michigan Medical School, Ann Arbor, Michigan, United States of America; 3 Department of Pathology, University of Massachusetts Medical School, Worcester, Massachusetts, United States of America; University of London, St George's, United Kingdom

## Abstract

Antigen cross-presentation involves the uptake and processing of exogenously derived antigens and their assembly with major histocompatibility complex (MHC) class I molecules. Antigen presenting cells (APC) load peptides derived from the exogenous antigens onto MHC class I molecules for presentation to CD8 T cells. Calreticulin has been suggested to mediate and enhance antigen cross-presentation of soluble and cell-derived antigens. In this study, we examined roles for calreticulin in cross-presentation of ovalbumin using a number of models. Our findings indicate that calreticulin does not enhance *in vitro* cross-presentation of an ovalbumin-derived peptide, or of fused or bead-associated ovalbumin. Additionally, *in vivo*, calreticulin fusion or co-conjugation does not enhance the efficiency of CD8 T cell activation by soluble or bead-associated ovalbumin either in wild type mice or in mice lacking Toll-like receptor 4 (TLR4). Furthermore, we detect no significant differences in cross-presentation efficiencies of glycosylated *vs.* non-glycosylated forms of ovalbumin. Together, these results point to the redundancies in pathways for uptake of soluble and bead-associated antigens.

## Introduction

Antigen presenting cells present peptides bound to MHC class I or class II molecules to T cells; this process facilitates T cell development, homeostasis, peripheral tolerance and activation of antigen specific T cells. Typically, MHC class I molecules present peptides derived from endogenous antigens to CD8 T cells. However, exogenous antigens can also be presented by MHC class I molecules of professional APCs such as dendritic cells (DC) by a process termed cross-presentation. APCs internalize extracellular soluble or cell-associated antigens and traffic the material to intracellular compartments that facilitate MHC class I presentation of the exogenously derived antigens (reviewed in [1]). Cross-presentation is suggested to be critical for the maintenance of CD8 T cell peripheral tolerance and for generation of cytotoxic T cell responses against intracellular pathogens and tumor cells (reviewed in [1]).

Calreticulin is an endoplasmic reticulum (ER)-localized chaperone that aids in the intracellular assembly of nascent MHC class I molecules and other newly synthesized glycoproteins (reviewed in [2]). Previous studies have also shown that calreticulin purified from tumor cells can elicit tumor-specific protective immunity [3], [4]. Calreticulin is a protein chaperone with glycoprotein and polypeptide-specific binding sites (reviewed in [2]). The immunogenic properties of purified tumor cell-derived calreticulin [3], [4] can be explained by co-purification with calreticulin of various tumor-derived peptides or proteins. Anti-tumor immunity conferred by purified calreticulin could derive from calreticulin-dependent delivery of intracellular antigens to relevant APCs. Alternatively or additionally, calreticulin-specific receptors could confer quantitative cross-presentation advantages. The latter possibility is suggested by findings that calreticulin cross-presents associated peptides more efficiently compared to the peptides alone *in vitro* and *in vivo* [3], [5].

CD91, scavenger receptor A (SRA), and scavenger receptor expressed by endothelial cell-I (SREC-1) are suggested to function as receptors for extracellular or cell-surface forms of calreticulin [5], [6], [7], [8]. These receptors are not exclusive to calreticulin as they bind other heat shock proteins such as gp96, HSP90 and HSP70 [5], [6], [9]. A particular receptor could quantitatively impact cross-presentation by specific enhancement in antigen uptake [10] or by directing antigen into distinct compartments favorable for cross-presentation [11]. Recent studies indicate that SRA deficiency enhances cross-presentation of cell-associated antigens and anti-tumor immunity [12], [13]. However, it is unknown whether other receptors implicated in binding and uptake of calreticulin (CD91, SREC-1 [5], [7], [8], or other uncharacterized receptors) can enhance cross-presentation efficiency of associated antigens. To address the contributions of any receptor system that may be relevant to uptake and cross-presentation of calreticulin-associated antigens, in this study, we examined relative cross-presentation efficiencies of a calreticulin-antigen fusion compared to antigen alone. For these studies, we used the model antigen ovalbumin expressed and purified from *E. coli*. The mannose receptor is a C-type lectin receptor suggested to be important for uptake and cross-presentation of ovalbumin [14], [15]. As the mannose receptor is expected to recognize glycan components of ovalbumin (reviewed in [16]), it was also of relevance to this study to compare cross-presentation efficiencies of the non-glycosylated *E. coli*-derived ovalbumin with that of glycosylated ovalbumin derived from a natural source, which was undertaken.

## Results

### Calreticulin does not enhance the cross-presentation of a peptide antigen

**Figure 1:**
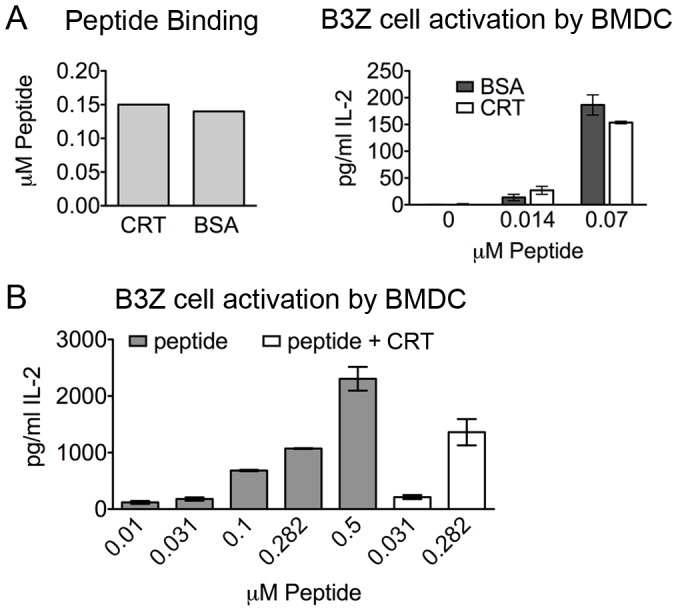
Cross-presentation of a peptide antigen. (A) 10 µM calreticulin (CRT) or bovine serum albumin (BSA) were incubated with 10 µM peptide (QLESIINFEKLTE-FITC). Free peptide was removed using a centrifugal filter device at 4°C, and peptide still in complex with CRT or BSA was measured (left panel). CRT- or BSA-peptide complexes were incubated with BMDC and B3Z cells. IL-2 production was determined by ELISA of the supernatants after 24 hours (right). Peptide concentration is indicated; CRT or BSA were present at a final concentration of 1 µM. (B) Cross-presentation of free peptide or CRT-peptide complexes was measured as in *A*. Data are representative of two independent analyses for both *A* (right panel) and *B*. Mean ± s.e.m. are shown in A and B.

Calreticulin-peptide complexes form when peptides and calreticulin are mixed and heat shocked to 50°C [3]. To examine whether calreticulin-specific receptors enhance the cross-presentation of a calreticulin-associated peptide, we heated calreticulin or bovine serum albumin (BSA) with a FITC labeled peptide derived from the model antigen OVA (amino acids 255–267: QLESIINFEKLTE-FITC) at 50°C for 1 hour. Free peptide was removed using a centrifugal filter device, and the amount of peptide bound to calreticulin and BSA was examined. We observed that the peptide bound equally to both calreticulin and BSA (Figure 1A, left panel). We next incubated bone marrow-derived dendritic cells (BMDC) with the peptide complexes or peptide alone, and then added to the culture a T cell hybridoma line (B3Z) whose T-cell receptor ligand is the OVA_258–265_ epitope (SIINFEKL) bound to the murine MHC class I allele H2-K^b^ [17]. We observed equal IL-2 levels in the culture supernatants when comparing responses to calreticulin-associated or BSA-associated peptide (Figure 1A, right panel) and equal IL-2 levels when comparing calreticulin-associated peptide or free peptide (Figure 1B). We also incubated the calreticulin- or BSA-peptide complexes with bone marrow-derived macrophages (BM Mφ), as Mφ express a different set of receptors than dendritic cells. Similar to the results seen with BMDC, calreticulin did not enhance the cross-presentation of a peptide-associated antigen compared to BSA when BM Mφ were used as the APC (data not shown). Thus, the calreticulin-specific receptors that have been reported [5], [6], [7] are not sufficient to enhance the cross-presentation of the peptide used in this study.

### Calreticulin does not enhance the cross-presentation of a fused protein antigen

**Figure 2:**
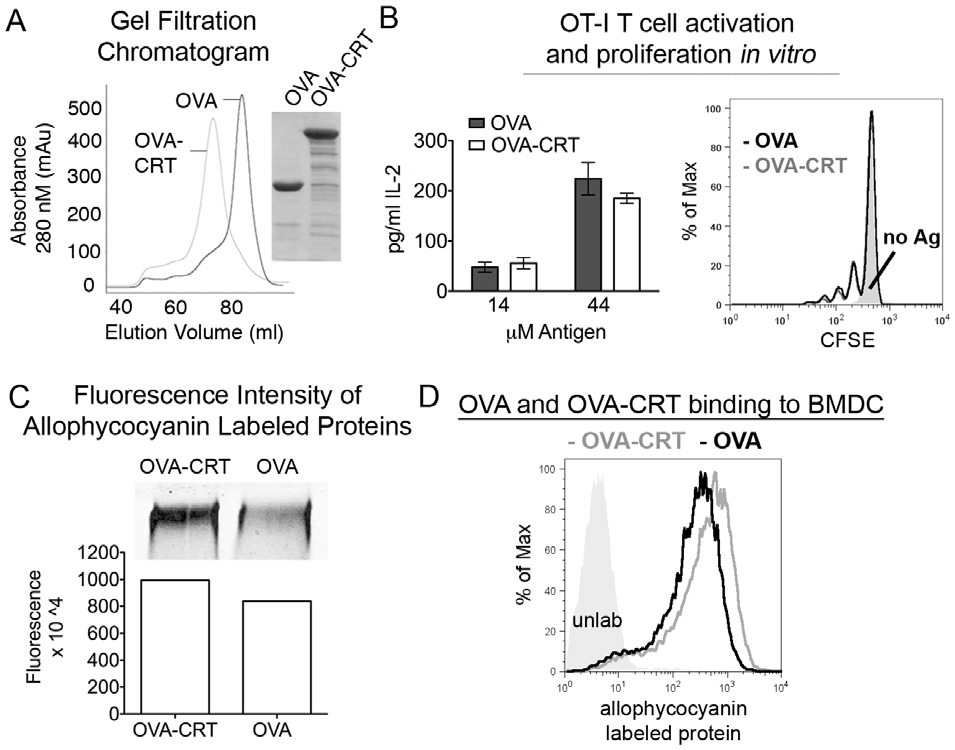
*In vitro* cross-presentation of a calreticulin-fused soluble antigen. (A) Gel-filtration chromatogram of *E. coli*-derived OVA or the OVA-calreticulin (OVA- CRT) fusion protein (left). SDS-PAGE analysis of pooled fractions from left panel; proteins were loaded in equimolar amounts (right) and coomassie stained. (B) Indicated proteins were incubated with BMDC for 3 hours. BMDC were fixed and CFSE labeled OT-I T cells were added. IL-2 levels in supernatants were determined by ELISA (left panel; 24 hour time point). OT-I T cell proliferation was measured at 72 hours in response to 44 µM OVA or OVA-CRT. The solid grey profile indicates the condition where no antigen (no Ag) was added. Data are representative of two independent analyses. (C, D) OVA-CRT and OVA were labeled with allophycocyanin. (C) Labeling intensity was determined by fluorescence imaging of the proteins after separation by SDS-PAGE (inset). Fluorescence intensity was quantified for the indicated proteins. (D) Binding of fluorescent proteins to BMDC was assessed by flow cytometry. BMDC were incubated with labeled proteins on ice before being analyzed by flow cytometry. BMDC not incubated with proteins are depicted as a grey filled. Representative of two independent experiments performed with the same labeled proteins. Mean ± s.e.m. are shown in *B*.

The extended peptide used in Figure 1 does not have high specificity for calreticulin binding, as similar amounts of peptide were recovered following heat shock with calreticulin or BSA (Figure 1A, left panel). The peptide-binding site of calreticulin and specificity of peptides that bind to calreticulin are poorly understood and defined. Hence, we generated a fusion protein between full-length OVA and calreticulin that linked OVA to the N-terminus of calreticulin (OVA-CRT). Both OVA-CRT and OVA were expressed in *E. coli* and contained a histidine tag for purification. Proteins were first purified over a nickel column and then further purified and analyzed on a size-exclusion column. OVA-CRT and OVA were both isolated predominantly as single peaks, results indicative of the homogeneity and stability of both proteins. The major peak of both proteins was isolated and used for subsequent experiments (Figure 2A).

To assess the cross-presentation efficiency of OVA-CRT compared to OVA, OVA-CRT or OVA were incubated with BMDC and CFSE labeled OT-I T cells (obtained from a transgenic mouse whose CD8 T cells express a T cell receptor that recognizes the OVA_257–264_ epitope [SIINFEKL] bound to the murine MHC class I allele H2-K^b^ [18]). Levels of IL-2 in the supernatant were measured after 24 hours and OT-I T cell proliferation was measured after 3 days. No significant differences were observed in levels of IL-2 produced by the OT-I T cells in response to OVA or OVA-CRT, and proliferation of the OT-I T cells was found to be very similar (Figure 2B). To assess binding of OVA and OVA-CRT to BMDC, both proteins were labeled with allophycocyanin. Higher level of fluorescence incorporation was observed for OVA-CRT compared to OVA, and correspondingly, binding to BMDC was slightly enhanced for OVA-CRT compared to OVA (Figure 2C and 2D). Due to the difficulty in achieving equivalent labeling of OVA-CRT and OVA, it remains unclear whether calreticulin fusion to OVA confers a specific BMDC binding advantage to OVA.

**Figure 3:**
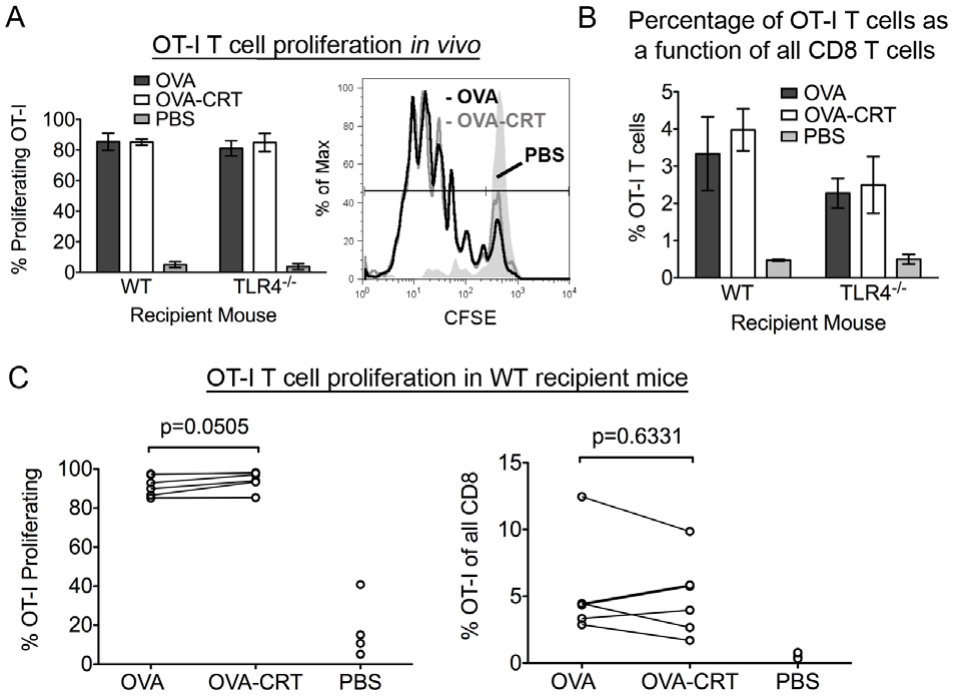
*In vivo* cross-presentation of a calreticulin-fused soluble antigen. (A, B) WT or TLR4^−/−^ recipient mice were injected i.v. with CFSE labeled OT-I T cells. Twenty-four hours later, mice received s.c. injections of the indicated antigen (100 µl of a 220 nM solution). OT-I T cell proliferation was measured 3 days later in the dLN (inguinal). (A) The % of proliferating OT-I T cells averaged from the mice of one experiment is shown in the left panel. Two to three mice were used in all groups. The right panel depicts proliferation in WT recipient mice. (B) Quantification of the % of OT-I T cells of all CD8 T cells recovered in *A*. Data for *A* and *B* are representative of three out of four independent analyses for WT recipients and a single analysis with TLR4^−/−^ recipients. Similar results were obtained in comparisons of OVA and OVA-CRT-induced OT-I proliferation in WT and TLR2/4^−/−^ recipient mice (data not shown). (C) Compilation of the % of proliferating OT-I T cells (left panel) and of the % of OT-I T cells as a function of all CD8 T cells (right panel) from 4 independent experiments performed with WT recipient mice. Two experiments contained 2 doses of antigen and two experiments contained 1 antigen dose. Antigen doses ranged from 0.22 µM–22 µM, using 100 µl. Each point represents the mean of 2–3 mice for that condition. Mean ± s.e.m. are shown in *A* and *B*. A two-tailed pair-wise student t-test was used for statistical analyses in *C*.

To evaluate the *in vivo* responses to the soluble proteins, CFSE labeled OT-I T cells were injected intravenously (i.v.) into wild-type (WT) recipient mice. Twenty-four hours later, mice were immunized subcutaneously (s.c.) with equimolar amounts of OVA or OVA-CRT. Three days after the immunization, proliferation of the OT-I T cells from the draining lymph node was assessed. Percentages of proliferating OT-I T cells (Figure 3A and 3C, left panel) and percentages of OT-I T cells recovered (Figure 3B and 3C, right panel) were similar in response to OVA, whether or not OVA was fused to calreticulin.

In the experiments described thus far, lipopolysaccharide (LPS) contamination was of concern because the recombinant proteins used in these experiments are of *E. coli* origin. As LPS is an agonist for TLR4 [19], it was conceivable that LPS contamination could mask potentiating effects of calreticulin. To address this concern, TLR4^−/−^ recipient mice [20] were used, previously characterized for their inability to respond to LPS. OVA-CRT did not induce a greater CD8 T cell response compared to OVA alone even when TLR4^−/−^ mice were used as recipients (Figure 3A, left panel and 3B). We concluded that there was no specific advantage for OT-I T cell proliferation when OVA was fused to calreticulin compared to OVA alone, even in the absence of LPS signaling. It is however noteworthy that percentages of OT-I T cells recovered in response to OVA and OVA-CRT were reduced in TLR4^−/−^ mice compared to WT mice (Figure 3B). This reduction was not surprising as TLR4 engagement on APCs induces APC maturation and migration to lymph nodes, resulting in augmented T cell responses (reviewed in [21]). These results are also consistent with previous findings that TLR signaling enhances *in vitro* cross-presentation of soluble antigen [22], and that LPS induces cross-presentation of ovalbumin, following intramuscular immunization [23].

### Calreticulin does not enhance the cross-presentation of a bead-associated antigen

**Figure 4:**
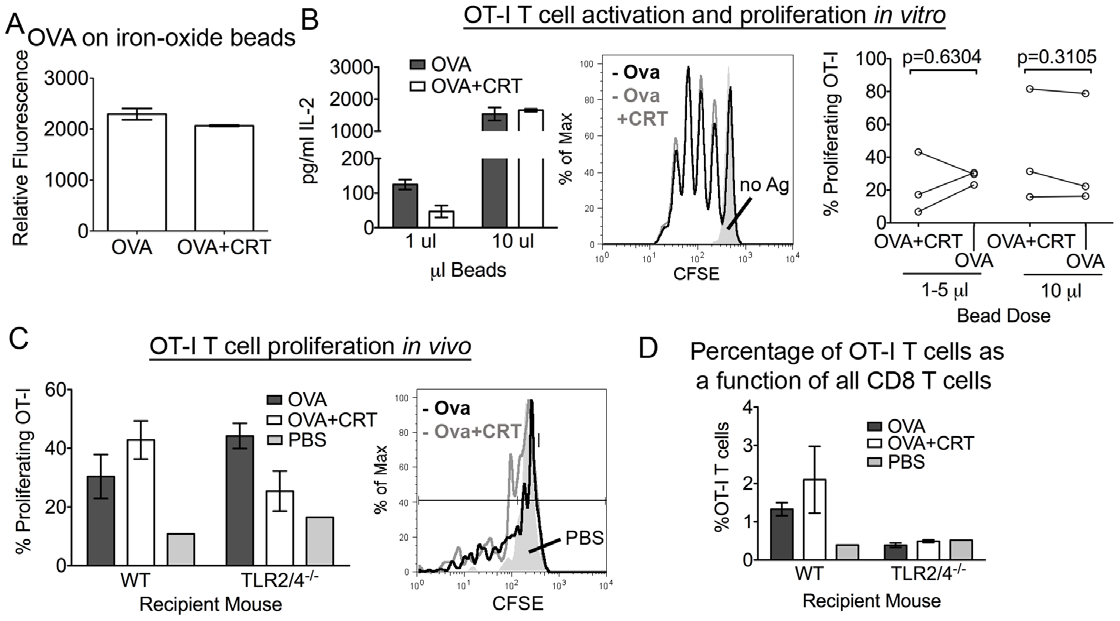
Cross-presentation of a particulate antigen. (A) OVA alone or with calreticulin (OVA+CRT) were conjugated to iron-oxide beads. Levels of OVA conjugated to the beads were quantified using a fluorescence-based assay. (B) The indicated beads were incubated with BMDC for 3 hours. BMDC were fixed and CFSE labeled OT-I T cells were added. Left and middle panels are representative of 1 independent experiment. IL-2 production was determined by ELISA of the supernatants at 24 hours (left panel). OT-I T cell proliferation was measured after 72 hours in response to 10 µl OVA or OVA+CRT beads. The solid grey profile indicates the condition where no antigen (no Ag) was added (middle panel). A compilation of the % of proliferating OT-I T cells from 3 independent experiments is depicted in the right panel in response to 1–5 or 10 µl beads. (C, D) OT-I T cell proliferation and recovery were measured in WT or TLR2/4^−/−^ mice in response to 50 µl beads on day 3. (C) Average proliferation values for 2–3 mice per group (except PBS, where 1 mouse was used) are shown in the left panel. A representative proliferation prolife from WT recipients in response to OVA beads, OVA+CRT beads or PBS (filled in grey) is shown on the right panel. (D) Quantification of the % of OT-I T cells of all CD8 T cells recovered in *C*. Data are representative of two independent analyses for *C*. Mean ± s.e.m. are shown in *A–D*. A two-tailed pair-wise student t-test was used for statistical analysis in *B*.

We next hypothesized that calreticulin may induce cross-presentation of a particulate antigen, as calreticulin has been shown to function as an “eat-me” signal when present on the surface of cells [8], [24], [25], [26]. OVA or an equimolar mixture of OVA and calreticulin (OVA+CRT) were conjugated to 1.5 µm iron oxide beads, and cross-presentation efficiencies were assessed *in vitro* and *in vivo*. Levels of OVA conjugated to beads were quantified with a fluorescent Ab. Comparable amounts of OVA were conjugated in the OVA and OVA+CRT bead preparations (Figure 4A). Similar to Figure 2B, OVA or OVA+CRT beads were next incubated with BMDC and CFSE labeled OT-I T cells. Again, there was no difference in the levels of IL-2 generated or in the proliferation of the OT-I T cells induced in response to OVA or OVA+CRT beads (Figure 4B). Similar results were obtained *in vivo* with WT or TLR2/4^−/−^ recipient mice [27] (Figure 4C and 4D). As noted above, percentages of OT-I T cells recovered in response to OVA and OVA+CRT beads were reduced in TLR2/4^−/−^ mice compared to WT mice (Figure 4D). Both sets of results echoed our findings using the soluble OVA-CRT fusion protein. Taken together, we concluded that extracellular calreticulin, in a soluble or particulate context, does not enhance the cross-presentation of associated antigen.

### Glycosylated and non-glycosylated OVA are cross-presented with similar efficiencies

Cross-presentation of soluble OVA is suggested to be mediated by the mannose receptor [14]. OVA purified from *E. coli* is not glycosylated, as *E. coli* lack eukaryotic glycosylation machinery. However, we found that non-glycosylated OVA isolated from *E. coli* was cross-presented *in vivo* (Figure 3), indicating that the mannose receptor may be non-essential for the cross-presentation of soluble OVA. This result prompted us to compare the *in vivo* cross-presentation efficiencies of OVA purified from *E. coli* and OVA obtained from Sigma [OVA (Sigma)], which is glycosylated as it is isolated from chicken eggs. This comparison would allow us to further determine the importance of OVA uptake through the mannose receptor or alternative pathways upon the proliferation of OT-I T cells.

**Figure 5:**
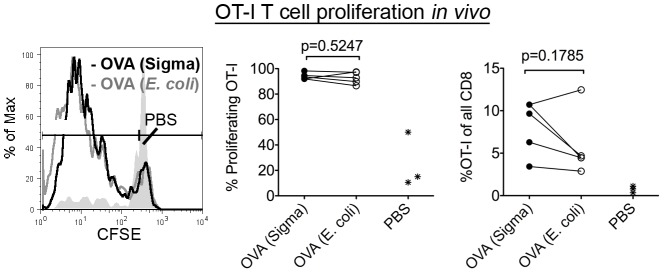
In vivo cross-presentation of glycosylated and non-glycosylated OVA. WT recipient mice were injected i.v. with CFSE labeled OT-I T cells. Twenty-four hours later, mice received s.c. injections of the indicated antigen (2.5–100 µg). OT-I T cell proliferation was measured 3–5 days later in the dLN (inguinal). Left panel: A representative proliferation profile is presented in response to 2.5 µg OVA. Middle and right panels: Two to three mice are averaged to generate each data point, which represents the % of proliferating OT-I T cells (middle) or the % of OT-I T cells as a function of all CD8 T cells recovered (right). Three independent experiments are represented. In one experiment, 2 different OVA (*E. coli*) preps were used in 2 different groups of mice. In another experiment, two doses (10 µg and 100 µg) of OVA were used in 2 different groups of mice. A two-tailed pair-wise student t-test was used for statistical analysis.

We assessed the abilities of s.c. delivered OVA (*E. coli*) and OVA (Sigma) to induce proliferation of CFSE labeled OT-I T cells *in vivo*. Using WT mice as recipients, the percentages of proliferating OT-I T cells were similar in response to OVA (Sigma) compared to OVA (*E. coli*) (Figure 5, left and middle panels; p = 0.5247). The percentage of OT-I T cells recovered was also similar in response to OVA (Sigma) compared to OVA (*E. coli*) (Figure 5, right panel; p = 0.1785), although there was a trend towards a stronger response with OVA (Sigma). We concluded that glycosylated OVA does not have a significant cross-presentation advantage compared to non-glycosylated OVA *in vivo*. Taken together, the data in Figure 5 suggest that the mannose receptor is non-essential for the cross-presentation of soluble OVA *in vivo*.

## Discussion

One previous study showed enhanced cross-presentation of a soluble peptide by Mφ following heat-shock in the presence of calreticulin, compared to peptide alone [5]. Using an extended version of the OVA-derived SIINFEKL peptide, we were unable to demonstrate enhanced calreticulin-dependent cross-presentation by BMDC or BM Mφ (data not shown). The discrepancies could reflect differences between antigens used in the two studies, and it was possible that the extended SIINFEKL peptide lacked adequate specificity for calreticulin. Rules for high affinity binding of particular peptide sequences to calreticulin are not well understood. By linking full-length OVA to calreticulin, we by-passed the need to identify a peptide with a high affinity for calreticulin. We were unable to observe enhanced CD8 T cell proliferation *in vitro* or *in vivo* in response to extracellular OVA-CRT compared to OVA alone (Figures 2 and 3). Previous studies indicate a significant but non-essential role for the mannose receptor in the uptake of ovalbumin [14], [15], although other studies suggest the requirement for mannose receptor may be APC-dependent [28]. The ovalbumin proteins used in Figures 2 and 3 were recombinant proteins of *E. coli* origin, and are thus not expected to be internalized via the mannose receptor due the absence of glycan modifications. Nonetheless, significant activation of the OT-I T cell response was induced *in vivo* following OVA or OVA-CRT s.c. immunizations at antigen doses as low as 1 µg/mouse (Figure 3C and replicate analyses). In the analyses of Figures 2 and 3, uptake of the OVA and OVA-CRT proteins may occur via receptor-mediated endocytosis that involves mannose receptor-independent uptake pathways, or via pinocytosis. This possibility is further supported by the analyses shown in Figure 5, where no significant differences were measurable between cross-presentation efficiencies of ovalbumin derived from *E.coli* or chicken eggs, which are expected to be non-glycosylated and glycosylated respectively. Altogether, the studies described in Figures 2, 3, and 5 taken together with previous studies [14], [15], [28] indicate that multiple uptake pathways can contribute to the cross-presentation of ovalbumin. Regardless of the precise uptake pathway, it is noteworthy that ovalbumin delivered as a calreticulin fusion does get cross-presented, although a specific cross-presentation advantage that results from the calreticulin fusion is not apparent. Thus, if calreticulin-specific receptors contribute to increased uptake of the OVA-CRT fusion *in vitro* or *in vivo*, such uptake does not result in increased cross-presentation. Taken together, our studies suggest that the immunogenic properties of calreticulin purified from tumor cells [3], [4] must result from co-purification and subsequent cross-presentation of one or more tumor-derived antigens, rather than a calreticulin-dependent influence on the cross-presentation pathway *per se*. By binding to antigen within its substrate binding site(s), calreticulin could protect antigen from complete proteolytic degradation, thus preserving antigen in a form that is competent for subsequent cross-presentation. General uptake pathways for soluble antigen may be operative during the cross-presentation of calreticulin-antigen complexes, and it is possible that calreticulin binding can confer a kinetic advantage for cross-presentation over complete degradation, at least for some antigens. The latter mechanism might explain previous findings of the potentiating activity of calreticulin during cross-presentation of elongated peptides [5].

It remains possible that the soluble OVA-CRT construct may not have been able to bind calreticulin-specific receptors with a high enough avidity to impact the cross-presentation of OVA. OVA may have masked the calreticulin-receptor binding site on calreticulin. To address this issue, calreticulin and OVA or OVA alone were conjugated to iron oxide beads and cross-presentation efficiencies were assessed. OVA and calreticulin are not fused in this system. Thus, calreticulin-receptor interactions should not be inhibited. We show that calreticulin was not able to enhance the cross-presentation of the bead-associated OVA compared to beads with OVA alone, both *in vitro* and *in vivo* (Figure 4). Calreticulin has been reported to be an “eat me” signal on the surface of apoptotic cells [8], [25]. The findings of Figure 4 suggest that calreticulin does not work independently in a phagocytic context, but rather might work in conjunction with other “eat me” signals such as phosphatidylserine. Hence, calreticulin in isolation is not sufficient to enhance cross-presentation of a particulate antigen. However, it is also possible that phagocytic uptake of the iron oxide beads is intrinsically high, even in the absence of calreticulin.

In summary, we have examined whether calreticulin can influence CD8 T cell proliferation against peptide, soluble and bead-associated antigen. We show that ovalbumin is cross-presented with similar efficiency when delivered alone compared to delivery with calreticulin as a soluble fusion, or co-conjugated on beads. Additionally, glycosylated and non-glycosylated forms of ovalbumin are cross-presented with similar efficiencies. TLR4 signaling induces efficiency of cross-presentation of subcutaneously delivered soluble and bead-associated antigens. Further studies are needed to understand roles for calreticulin and relevant receptors in phagocytosis and cross-presentation in the context of cell-associated antigens and subcutaneous immunizations.

## Materials and Methods

### Mice

All mice were maintained in specific pathogen-free conditions at the University of Michigan or the University of Massachusetts Medical School (UMMS) mouse facilities. All experiments involving mice were approved by and performed in accordance with guidelines set forth by the University Committee on Use and Care of Animals (UCUCA) at the University of Michigan or the UMMS Department of Animal Medicine and the Institutional Animal Care and Use Committee. C57BL/6J (WT or B6 in text; CD45.2), B6.SJL-*Ptprc^a^ Pepc^b^*/BoyJ (WT or B6 in text; CD45.1), and C57BL/6-Tg(TcraTcrb)1100Mjb/J (OT-I in text) mice were purchased from The Jackson Laboratory. OT-I transgenic mice were used directly (CD45.2) or bred with B6.SJL-Ptprca Pep3b/Boy mice (The Jackson Laboratory) to yield CD45.1 T cells. TLR2^−/−^ and TLR4^−/−^ mice [20], [27] were provided by Dr. S. Akira at the Laboratory of Host Defense, Osaka University, Osaka, Japan. TLR2/4^−/−^ mice were generated by crossing TLR2^−/−^ with TLR4^−/−^ mice. TLR4^−/−^ mice were backcrossed onto the B6 background six times before being bred with TLR2^−/−^ mice.

### Cell culture, purification and labeling

#### Cell lines

The B3Z hybridoma T cell line [17] was maintained in RPMI+ [RPMI medium 1640 (Invitrogen) supplemented with 10% (v/v) fetal bovine serum, 100 µg/ml streptomycin, and 100 units/ml penicillin (Invitrogen)]. Cells were maintained in an incubator kept at 37°C with 5% CO_2_.

#### BMDC

Bone marrow was flushed from the femur and tibia with RPMI+. The red blood cells were lysed using red cell lysis buffer (Sigma), and the cells were re-suspended in RPMI medium supplemented with 10% (v/v) fetal bovine serum (Gibco), 100 µg/ml streptomycin, 100 units/ml penicillin (Invitrogen), 1 mM HEPES (Gibco), 0.1 mM MEM Non-Essential Amino Acids (Gibco), 1 mM Sodium pyruvate, 50 µM β-mercaptoethanol and granulocyte Mφ colony-stimulating factor (GM-CSF). The bone marrow obtained from one mouse was plated into two 24-well plates (Corning). The medium was replaced on days 2 and 4, and the cells were harvested for *in vitro* experiments on day 5 or 6.

#### Purification of splenic CD8+ T cells

Spleens were extracted from OT-I transgenic mice. The red blood cells were lysed using red cell lysis buffer (Sigma), and the CD8^+^ cells were isolated by positive selection using anti-CD8a (Ly-2) microbeads (MACS, Miltenyi Biotec), respectively, following the manufacturer's suggested protocol.

#### CFSE labeling

MACS purified CD8+ T cells from OT-I transgenic mice were labeled with CFSE for proliferation analyses. CD8+ T cells were washed once with PBS, centrifuged, and re-suspended in PBS + 5 µM CFSE. The cells were incubated at 37°C for 10 min. The cells were washed once with medium, re-suspended in RPMI+ and incubated at 37°C, 5% CO_2_ for 1 hour. The cells were then centrifuged and re-suspended in an appropriate volume of RPMI+ for *in vitro* experiments or PBS for *in vivo* experiments. On average, 3–5×10^6^ live, CFSE labeled, CD8+ OT-I T cells were recovered from one spleen, where trypan blue staining assessed viability.

### DNA constructs

#### Expression of calreticulin, OVA, and OVA-CRT in *Escherichia coli*

Mouse calreticulin (accession number BC003453) was amplified from the pCMV-SPORT6 (ATCC, MGC-6209) vector using primers that allowed for subsequent ligation-independent cloning (LIC) into the pMCSG7 vector [29]. The following primers were used: forward, 5′ TAC TTC CAA TCC AAT GCT GCC GCA CAT CCT TGG CTT 3′ and reverse, 5′ TTA TCC ACT TCC AAT GTT ACA GCT CAT CCT TGG CTT 3′. Underlined bases represent those that are complementary to the sequence encoding calreticulin, and additional 5′ sequences were introduced for LIC.

Chicken egg OVA (accession number V00383) was amplified for LIC. The following primers were used: forward: 5′ TAC TTC CAA TCC AAT GCT ATG GGC TCC ATC GGC G 3′ and reverse, 5′ TTA TCC ACT TCC AAT GTT AAG GGG AAA CAC ATC TGC 3′. Underlined bases represent those that are complementary to the sequence encoding OVA, and additional 5′ sequences were introduced for LIC.

The OVA-CRT fusion protein was constructed using a 2-step amplification process resulting in a full length OVA molecule fused to the N-terminus of full length calreticulin by a flexible linker (gly-gly-ser-gly-gly). The reverse OVA primer was complementary to the forward calreticulin primer, allowing for the fusion of the two PCR products in a second PCR reaction. OVA was amplified using the following primers: 5′ TAC TTC CAA TCC AAT GCT ATG GGC TCC ATC GGC G 3′ and reverse, 5′ *GGC AGG GTC TGC GGC* **TCC TCC TGA TCC ACC** AGG GGA AAC ACA TCT 3′. Calreticulin was amplified using the following primers: forward, 5′ AGA TGT GTT TCC CCT **GGT GGA TCA GGA GGA** *GCC GCA GAC CCT GCC* 3′ and reverse, 5′ TTA TCC ACT TCC AAT GTT *ACA GCT CAT CCT TGG CTT* 3′. Underlined bases represent those that are complementary to the sequence encoding OVA, bold bases represent the introduced linker sequences (gly-gly-ser-gly-gly), italicized bases represent those that are complementary to calreticulin, and additional sequences were introduced for LIC. Both PCR products were run on a 0.8% agarose gel and gel-purified (Qiagen). Both products were then used together as templates in a second PCR reaction using the forward OVA primer and reverse calreticulin primer, which allowed for subsequent LIC of the OVA-CRT fusion into the pMCSG7 vector.

LIC was performed to introduce calreticulin, OVA, and OVA-CRT sequences into the pMCSG7 vector as previously described [30].

DNA constructs were sequenced and transformed into BL21 (DE3) cells for protein expression. All bacterially expressed constructs lacked their signal sequence and contained an N-terminal MHHHHHHSSGVDLGTENLYFGSNA fusion sequence for nickel affinity chromatography.

### Protein purification

Glycerol stocks of BL21 (DE3) cells expressing calreticulin, OVA, or OVA-CRT were inoculated into a 25-ml terrific broth culture (with 50 µg/ml ampicillin) and incubated at 37°C overnight. The starter culture was added to 1 liter of terrific broth with 50 µg/ml ampicillin and incubated at 37°C until cell density measured by A_600_ was 0.8–0.9. Cultures were then incubated at room temperature for 1 h before inducing calreticulin protein expression with 200 µM isopropyl 1-thio-ß-D-galactopyranoside. Bacterial cultures were incubated at room temperature for 16–20 h before harvesting cells by centrifugation. Cell pellets were resuspended in 50 ml of 50 mM Tris with 0.33 mg/ml lysozyme and EDTA-free complete protease inhibitors (Roche Applied Science). Cells were lysed by sonication. Subsequently, 10 µg/ml DNase, 1% Triton X-100, 10 mM MgCl_2_, and 1 mM CaCl_2_ were added, and the cell lysis suspension was incubated at room temperature for 30 min. Cell debris was removed from samples by centrifugation, followed by vacuum filtration of the supernatant with a Steriflip having a 0.22-µm pore membrane (Millipore). The filtrate was then incubated with nickel-nitrilotriacetic acid-agarose beads (Qiagen) for 2–4 h at 4°C. The beads were washed with 10 mM imidazole in wash buffer (50 mM Tris, 150 mM NaCl, 1 mM CaCl_2_, pH 7.5). Protein was eluted from beads with 75 and 100 mM imidazole in wash buffer. Protein was concentrated to 0.5–4.0 ml, by centrifugation using Centriplus centrifugal filter devices (Millipore) with molecular weight cut-offs of 10 or 30 kDa, and analyzed by gel-filtration chromatography.

Purified concentrated protein was analyzed by gel filtration at 4°C using a Superdex 200 10/300 GL column or Highload 16/60 Superdex 200 (Amersham Biosciences). Buffer used was 20 mM Hepes, 150 mM NaCl, 10% glycerol, 1 mM CaCl_2_, pH 7.5. Following gel filtration, fractions were pooled and concentrated by centrifugation using Centricon centrifugal filter devices (Millipore) with molecular weight cut-offs of 10 or 30 kDa. Protein concentration was determined measuring absorbance at 280 nm. Extinction coefficients were calculated from the protein amino acid sequence using ProtParam (www.expasy.ch) and are as follows (units are in M_−1_ cm_−1_): Calreticulin, 82,975; OVA, 33,265; OVA-CRT, 114,750.

### Generation of calreticulin-peptide and BSA-peptide complexes

10 µM calreticulin or BSA were incubated with 10 or 100 µM peptide (QLESIINFEKLTE-FITC; University of Michigan Protein Structure Facility) for 1 hour at 50°C. Free peptide was removed by centrifugal filter (Centricon-30, Millipore). Peptide bound to calreticulin or BSA was measured using a VICTOR plate reader (fluorimeter; PerkinElmer) against a peptide standard.

### OVA and OVA-CRT allophycocyanin conjugation

Allophycocyanin was conjugated to OVA and OVA-CRT following manufacturer's instructions at a 1∶1 molar ratio (Lightning-Link Conjugation Kit, Innova Biosciences). To measure fluorescence intensity, OVA and OVA-CRT were separated by SDS-PAGE and fluorescence was measured using a fluorescence imager (Typhoon TRIO, GE Healthcare). Quantification was performed using ImageQuant5.2 software.

### *In vitro* antigen cross-presentation assays

#### Assays using peptide antigen

3–5×10^4^ BMDC and 2–3×10^4^ B3Z cells were plated in a 96 well plate. Calreticulin-peptide complexes, BSA-peptide complexes, or peptide alone were added to the wells. At 24 hours, supernatant IL-2 levels were measured by ELISA.

#### Assays using soluble or bead-associated antigen

4–5×10^5^ BMDC were plated in a 96 well plate. Soluble proteins were then added to the wells. The cells were incubated at 37°C for 3 hours. BMDC were fixed with 1% formaldehyde for 7 min at room temperature. The wells were then washed two times with 200 µl PBS and once with RPMI+. Medium was aspirated from the wells, and a suspension of 4–5×10^5^ B3Z or 1–3×10^5^ CFSE labeled OT-I T cells in 250 µl RPMI+ was added to the wells. At 24 hours, supernatant IL-2 levels were measured by ELISA, and proliferation of OT-I T cells was measured at 72 hours by flow cytometry. OVA (Sigma) was obtained from Sigma (A5503).

### *In vivo* antigen cross-presentation assay

CD8+ OT-I T cells were MACS purified from CD45.1 or CD45.2 mice and transferred i.v. into CD45.2 or CD45.1 recipient mice, respectively. One day later, mice were immunized s.c. in either the left or right flank with the indicated concentration/amount of soluble or bead-associated antigen in 100 µL PBS. Three to four days later, draining lymph nodes (inguinal) were harvested and analyzed by flow cytometry.

### Flow Cytometry

#### OVA and OVA-CRT binding to BMDC

1×10^5^ BMDC were incubated with 1.3 µg OVA-CRT or 0.6 µg OVA that were allophycocyanin labeled in a total reaction volume of 100 µl for 30 minutes on ice. Cells were washed twice with flow cytometry buffer and analyzed by flow cytometry.

#### Detection of proliferating OT-I T cells

Draining lymph nodes (inguinal) were harvested; a single cell suspension was obtained and plated in a 96-well plate. Cells were lysed in red cell lysis buffer (Sigma). These cells were washed once with flow cytometry buffer (2% FBS in PBS) and then incubated for 10 min with unconjugated murine IgG (Jackson ImmunoResearch) to bind and block F_c_ receptors. Cells from draining lymph nodes or from *in vitro* cell cultures were stained with a rat anti-mouse CD8a Ab conjugated to allophycocyanin (BD Pharmingen, 1∶400) or anti-CD8a Ab conjugated to PerCP (eBioscience) and mouse anti-mouse CD45.1 or CD45.2 Ab conjugated to PE or allophycocyanin for *in vivo* experiments (BD Pharmingen, 1∶300 and eBioscience). Either CD45.1 or CD45.2 OT-I mice were utilized for *in vivo* adoptive transfers into WT strains (CD45.2 or CD45.1, respectively); the appropriate antibody was used to discriminate between donor and recipient cells. The cells were incubated with the Ab for 20 min, washed twice with flow cytometry buffer and then analyzed by flow cytometry. CFSE labeled OT-I T cells were analyzed in the FITC channel. All centrifugations in the 96-well plate were performed for 1 minute at 2,000 rpm.

### IL-2 ELISA

96-well plates were coated with purified anti-mouse IL-2 Ab (BD Pharmingen, catalog # 554424) overnight at room temperature. After blocking with 10% calf serum for 3 hours, cell culture supernatants were incubated in the plate for 1 hour at 37°C. The plates were washed 3 times with 0.05% Tween-20 in PBS and a biotinylated anti-mouse IL-2 Ab (BD Pharmingen, catalog # 554426) was added for an overnight incubation at room temperature. The plates were washed 3 times, and streptavidin conjugated to HRP (BD Pharmingen, catalog # 554066) was added to the plates for 20 min at room temperature. The plates were again washed 3 times, and the assay was developed using a TMB substrate reagent set (BD OptEIA, catalog # 555214).
